# Medical care costs at the end of life among older adults with cancer: a national health insurance data-based cohort study

**DOI:** 10.1186/s12904-023-01197-2

**Published:** 2023-06-23

**Authors:** Minjeong Jo, Yunji Lee, Taehwa Kim

**Affiliations:** 1 Research Institute for Hospice/Palliative Care, College of Nursing, The Catholic University of Korea, Seoul, Republic of Korea; 2 College of Nursing, Pusan National University, Yangsan, South Korea; 3 Division of Allergy, Pulmonary, and Critical Care Medicine, Department of Internal Medicine, Transplant Research Center, Research Institute for Convergence of Biomedical Science and Technology, Pusan National University Yangsan Hospital, Geumo-ro 20, Beomeo-ri, Mulgeum-eup, Yangsan-si, Gyeongsangnam-do 50612 Republic of Korea; 4 Department of Internal Medicine, Pusan National University School of Medicine, Busan, Republic of Korea

**Keywords:** Cancer, End-of-life care, Elderly, Medical care costs

## Abstract

**Objective:**

Along with aging, the elderly population with cancers is increasing. The costs of end-of-life (EOL) care are particularly high among cancer patients. The purpose of this study was to investigate the trends in medical costs in the last year of life among older adults with cancer.

**Design, setting, and participants:**

Using the Health Insurance Review and Assessment Services (HIRA) database for the period 2016–2019, we identified older adults aged ≥ 65 years who had a primary diagnosis of cancers and high-intensity treatment at least once in the intensive care unit (ICU) of tertiary hospitals.

**Main outcomes and measures:**

High-intensity treatment was defined as receiving at least one of the following treatments: cardiopulmonary resuscitation, mechanical ventilation, extracorporeal membrane oxygenation, hemodialysis, and transfusion. The EOL medical treatment costs were calculated by dividing periods 1, 2, 3, 6, and 12 months from the time of death, respectively.

**Results:**

The mean total EOL medical expense per older adult during the year before death was $33,712. The cost of EOL medical expenses for three months and one month before subjects’ death accounted for 62.6% ($21,117) and 33.8% ($11,389) of total EOL costs, respectively. Among subjects who died while receiving high-intensity treatment in the ICU, the costs associated with medical treatments that occurred during the last month before death were 42.4% ($13,841) of the total EOL expenses during the year.

**Conclusion:**

The findings indicate that EOL care expenditures for the older population with cancer are highly concentrated until the last month. The intensity of medical care is an important and challenging issue in terms of care quality and cost suitability. Efforts are needed to properly use medical resources and provide optimal EOL care for older adults with cancer.

## Introduction

Cancer is a leading cause of death; in 2019, cancer accounted for 27.5% of deaths in South Korea [1]. In particular, among the elderly population over 65 years, the mortality rate from cancer has continuously increased from 122.4 (per 100,000 population) in 2000 to 158.2 in 2019 [2]. Cancer patients are treated with multiple drugs and procedures for the management of symptoms and comorbid conditions. Additionally, a considerable proportion of cancer patients continue to receive intensive care near their end of life (EOL) [3]. South Korea has a national health insurance system that covers approximately 98% of the overall Korean population, and most medical procedures are reimbursed on a fee-for-service basis [4]. Under such circumstances, cancer-related medical expenditures can be a huge burden on national health insurance finances.

The pain and disability caused by disease can increase the burden on patients and their family members for a long time before death, not only physically and mentally, but also economically. In 2018, the proportion of medical expenditure spent by Korea in gross domestic product (GDP) was 7.6%. Although this proportion is lower than the average one of organization for economic co-operation and development (OECD, 8.8%), the rate of increase in medical expenditure over the past five years in South Korea is the fastest among OECD countries [5]. Medical expenses for the elderly ≥ 65 years continue to increase, and medical expenses per elderly person were approximately three times higher than that of the entire population in 2018 [2]. These data show that a surge in medical expenses due to an aging population will increase the burden on both individuals and national health insurance finances.

Given the high risk of morbidity and increased access to health care services, it is not surprising that healthcare costs increase with age. However, in addition to age, income, residence area, type of disease, and care at the EOL are significant factors contributing to the increase in medical expenses [6, 7]. In particular, there has been an increasing number of recent studies reporting medical expenses, which are intensively invested at the EOL and impending death, as a factor in the surge in medical expenses [3, 8]. In a Canadian cohort study [8], approximately 10% of government-funded health expenditures were spent on health care in the last year of life, and EOL care costs rose sharply in the last three months prior to death. The costs of EOL care are particularly high among patients with cancer. The cancer cohort had significantly higher total health care costs of EOL than those without cancer in the United States and Australia [6, 9].

Understanding EOL care costs can provide preliminary data on the factors that can increase the burden on the national health care budget. However, little is known about recent trends in medical care costs at the EOL among cancer patients in South Korea. Therefore, this study aimed to investigate the trends in medical costs for the last year of life among older adults with cancer.

## Methods

### Data sources

This study was a cohort study using data collected from the Health Insurance Review and Assessment Service (HIRA) database for the period 2016–2019. The HIRA database contains health insurance claims data from South Korea. The data include cost information of all medical services and prescription drugs that are reimbursed by the Korean National Health Insurance (KNHI) and the Medical Aid Program (MAP) [4]. The KNHI covers approximately 98% Koreans, and the MAP covers the remaining who cannot afford national insurance [4].

### Patient and public involvement

There was no direct patient or public involvement in this study. The study procedure was approved by the Institutional Review Board of the first author’s university in South Korea (PNU IRB/2021_53_HR).

### Study patient identification

Patients were considered for inclusion if they had a medical claim with a cancer diagnosis between 2016 and 2019. For this study, we extracted data of adults aged ≥ 65 years who had a primary diagnosis of cancer and high-intensity treatment at least once in the intensive care unit (ICU) of tertiary hospitals. Data from the HIRA were anonymized before providing them to the researchers.

### Study outcomes

#### Patients

Information on demographic characteristics, diagnoses, and health insurance was based on claims codes in the HIRA database. We identified older adults with one of the 12 cancers associated with high mortality in South Korea as the primary diagnosis [10]. The 12 cancers were: lung (C33 and C34), liver (C22), colorectal (C18-21), gastric (C16), gallbladder and biliary duct (C23, C24), pancreatic (C25), bladder (C67), prostate (C61), renal (C64, C65), breast (C50), thyroid (C73), and cervical (C53-55). Diagnoses were coded based on the Korean Standard Classification of Disease 7th editions [11], which is a modified version of the International Classification of Disease-10th revision (ICD-10) [12].

#### Medical care costs

The total costs of medical treatments were the sum of the reimbursed expenses by KNHI and MAP for the data extracted from one recent claim at the time of death or last hospitalization between 2016 and 2019 among older adults with cancer. Additionally, total costs for high-intensity medical treatments were calculated from these claims using the following procedure codes: cardiopulmonary resuscitation (CPR), mechanical ventilation (MV), extracorporeal membrane oxygenation (ECMO), hemodialysis (HD), and transfusion. We extracted the claim data that simultaneously included at least one high-intensity treatment code and ICU admission code. High-intensity treatment in this study was defined as receiving at least one of the following treatments in the ICU: CPR, MV, ECMO, HD, and transfusion. These are described as life-sustaining treatments in the Act of Hospice and Palliative Care and Decisions on Life-Sustaining Treatment for Patients at the End of Life (LST Decision Act) in South Korea [13]. In a Korean cohort study [14], the most commonly used high-intensity EOL care was transfusion, MV, and HD among deceased older adults. High-intensity treatments corresponded to the following codes based on the HIRA and KNHI [4]: CPR (KNHI codes M1583-7), MV (M5850-8, M5860), ECMO (O1901-6), HD (O7020 [intermittent hemodialysis; IDH] and O7031-4 [continuous renal replacement therapy; CRRT]), and transfusion (X2011-2, X2021-2, X2031-2, X2041-2, X2051-2, X2061-2, X2071-2, X2081-2, X2091-2, X2101-2, X2111-2, X2121-2, X2131-2, X2141-2).

#### EOL costs of medical treatments

The total EOL costs of medical treatments were defined as the sum of the reimbursed expenses by KNHI and MAP for the data extracted from claims during the previous year based on the time of death in deceased subjects. Medical expenses include outpatient and inpatient treatment, and patients pay only 5% of the total amount incurred in the hospital. This is in accordance with the national insurance policy for severe diseases in the Republic of Korea. To identify EOL treatment costs, we extracted claims data after 2017. The EOL medical treatment costs were calculated by dividing periods into 1, 2, 3, 6, and 12 months from the time of death.

### Statistical analysis

The data were analyzed using SAS version 9.4 (SAS Institute Inc., Cary, NC, USA). Descriptive statistics, including frequency, means, standard deviations (SD), and percentages, were computed to summarize the study population and medical treatment costs. Mean and SD were used to describe continuous variables, such as sample age and medical treatment costs. The costs for medical treatments in Korean won were converted to US dollars using a conversion rate of 1,200 won/dollar (exchange rates as of September 15, 2020). Additionally, a t-test or one-way ANOVA was conducted to investigate whether there were differences in costs according to demographic characteristics and high-intensity treatment type. The cost of ECMO was excluded from this analysis because it accounted for a very small proportion (0.23%) of the total medical care costs.

## Results

### Patient characteristics

The characteristics of the study sample are presented in Table 1. The mean age was 73.6 years (*SD* = 6.15), and 70.9% were men. Lung cancer was the most common diagnosis among subjects (32.0%), followed by liver (15.4%), colorectal (14.3%), gastric (10.15%), and gallbladder and biliary duct cancer (9.5%).

**Table 1:** Characteristics of study subjects (N = 6,098)

Characteristics	Categories	n	(%)	Medical care costs				Costs of high-intensity treatments		
				M ± SD	t or F	*p*		M ± SD	t or F	*p*
Overall				14029.09 ± 12351.61				849.30 ± 1696.35		
Age (years)	65-69^a^	2015	(33.0)	14509.06 ± 13754.54	6.286	< 0.001		1022.25 ± 1915.61	10.490	< 0.001
	70-74^b^	1723	(28.3)	13893.22 ± 12165.09		e < a		903.65 ± 1690.95		e < a
	75-79^c^	1397	(22.9)	13174.38 ± 10410.21				797.56 ± 1415.94		
	80-84^d^	700	(11.5)	13290.45 ± 11359.60				765.67 ± 1363.04		
	≥ 85^e^	263	(4.3)	11527.59 ± 9606.35				532.76 ± 1118.03		
Sex	Male	4326	(70.9)	14036.07 ± 12414.62	3.742	< 0.001		844.12 ± 1649.07	-2.462	0.003
	Female	1772	(29.1)	13212.25 ± 11897.87				919.28 ± 1715.09		
Health insurance	Medicare	5774	(94.7)	13791.74 ± 12239.64	2.753	0.001		885.72 ± 1672.51	− 0.670	0.126
	Medicaid	324	(5.3)	13209.85 ± 11083.89				904.15 ± 1453.35		
Diagnoses	Lung	1954	(32.0)	13352.25 ± 10357.22				537.32 ± 1037.42		
	Liver	940	(15.4)	15003.94 ± 17193.94				1456.94 ± 2354.25		
	Colorectal	873	(14.3)	13933.02 ± 11563.89				956.99 ± 1700.19		
	Gastric	614	(10.1)	14077.59 ± 12215.79				970.65 ± 1815.50		
	Gallbladder, Biliary duct	577	(9.5)	15093.16 ± 12669.43				965.74 ± 1672.91		
	Pancreas	417	(6.8)	11758.42 ± 8908.67				705.69 ± 1308.99		
	Bladder	207	(3.4)	12983.22 ± 10592.49				1023.67 ± 2058.27		
	Prostate	182	(3.0)	11430.34 ± 8818.27				918.31 ± 1480.08		
	Renal	112	(1.8)	14156.77 ± 11778.34				1083.32 ± 1716.27		
	Breast	107	(1.8)	13476.95 ± 12739.97				927.17 ± 1794.45		
	Thyroid	58	(1.0)	15093.33 ± 8797.26				709.61 ± 835.20		
	Cervical	57	(0.9)	11042.68 ± 7959.04				1122.30 ± 1322.62		

### Medical care costs

**Fig. 1:**
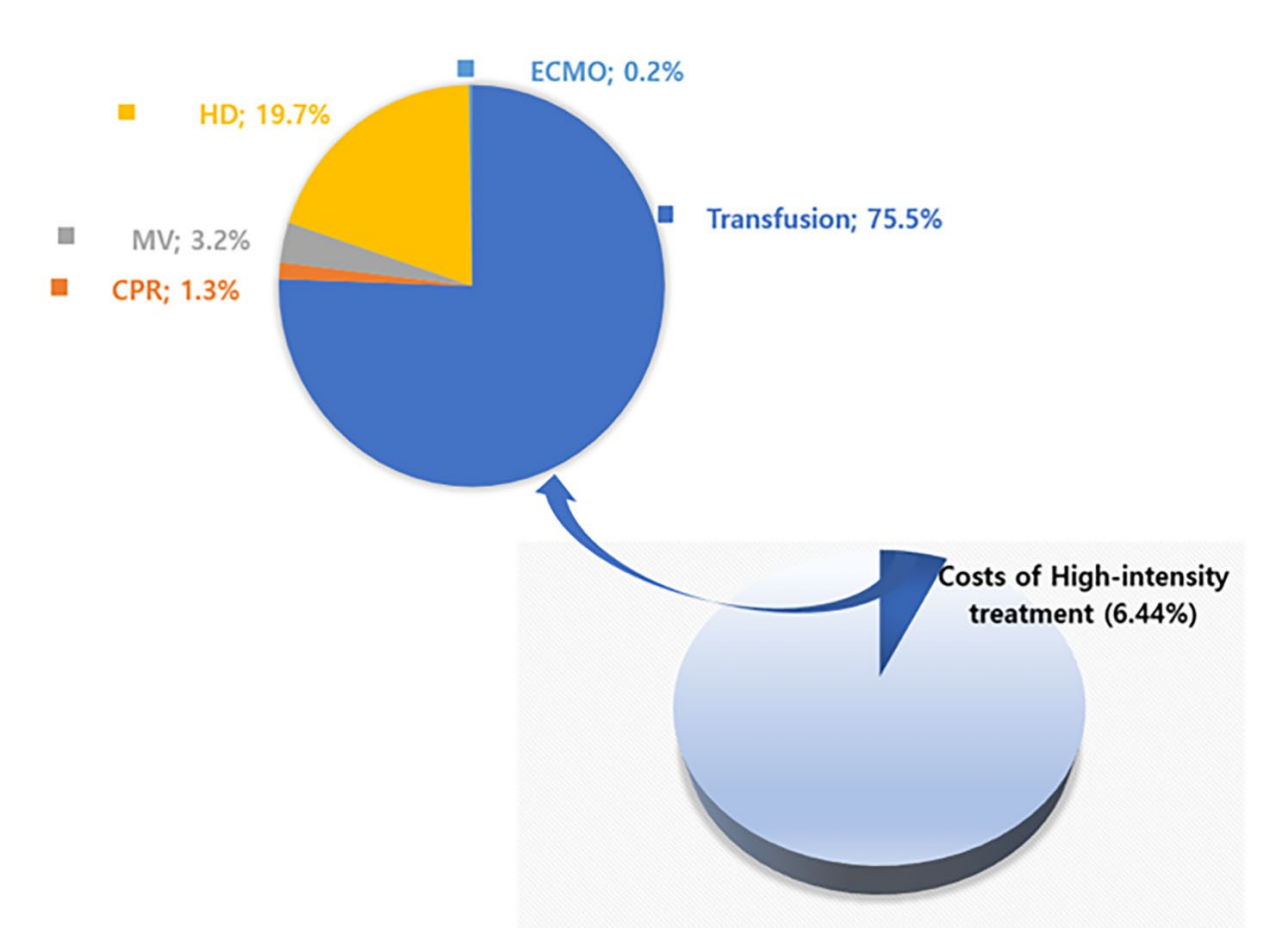
Proportion of costs associated with high-intensity treatment

As a result of analysis of subjects’ recent claim data during their hospitalization, the mean total medical treatment costs were $14,029.09 per patient and the mean total costs associated with high-intensity medical treatment was $849.30 (Table 1). High-intensity medical treatment costs accounted for 6.4% of the total costs (Fig. 1). Among the costs of high-intensity medical treatments, transfusion was the highest (75.5%), followed by HD (19.7%), and MV (3.2%). Both the mean costs of total medical treatments and high-intensity medical treatments were significantly higher in the 65 to 69 elderly group than in the ≥ 85 years group (Table 1, *p* < .001). The mean total medical treatment costs of subjects with KNHI were significantly higher than of those with MAP (*p* = .001)). The mean total medical treatment costs were significantly higher in men (*p* < .001), and the cost of high-intensity medical treatment was significantly higher in women (*p* = .003). For high-intensity medical treatment costs between men and women, there were differences according to treatment type (Table 2). The transfusion costs were likely to be higher in women, but these were higher for men diagnosed with gastric (*p* < .001) and gallbladder and biliary duct cancer (*p* = .015).

**Table 2:** Costs of high-intensity treatments between male and female (N = 6,098)

Variables		Male (M ± SD)					Female (M ± SD)					Compare (*p*)			
		Transfusion	CPR	MV	HD		Transfusion	CPR	MV	HD		Transfusion	CPR	MV	HD
Age (years)															
65–69		1008.79 ± 1791.80	123.39 ± 62.50	72.86 ± 66.44	826.30 ± 800.51		1147.74 ± 1978.26	113.59 ± 69.47	72.74 ± 49.07	775.98 ± 736.40		0.005	< 0.001	0.237	0.009
70–74		873.67 ± 1488.34	125.34 ± 56.69	69.67 ± 48.60	784.87 ± 696.03		1033.14 ± 1692.26	112.52 ± 41.88	76.05 ± 62.84	823.46 ± 790.90		0.001	< 0.001	< 0.001	0.032
75–79		747.05 ± 1238.92	122.18 ± 59.04	77.56 ± 113.97	752.01 ± 683.10		843.04 ± 1428.46	123.37 ± 78.39	67.65 ± 41.77	708.94 ± 594.93		0.014	0.163	0.001	0.019
80–84		787.94 ± 1365.42	119.17 ± 49.20	72.19 ± 52.98	773.63 ± 742.11		616.02 ± 951.65	118.67 ± 37.26	67.48 ± 43.70	757.54 ± 785.82		0.002	0.208	0.018	0.173
85-		549.06 ± 1336.80	119.92 ± 61.08	114.51 ± 202.16	600.50 ± 372.09		472.90 ± 738.65	120.52 ± 76.50	54.19 ± 32.61	646.18 ± 515.61		0.105	0.230	< 0.001	0.061
Health insurance															
Medicare		873.18 ± 1554.56	123.15 ± 59.20	74.16 ± 82.42	785.17 ± 737.59		938.75 ± 1632.40	115.27 ± 61.38	70.86 ± 51.39	750.19 ± 707.52		0.007	< 0.001	0.002	0.002
Medicaid		851.76 ± 1173.39	121.33 ± 43.84	71.20 ± 38.71	800.27 ± 599.21		819.12 ± 1400.37	135.15 ± 58.88	72.78 ± 45.88	956.45 ± 834.77		0.187	< 0.001	0.159	0.002
Diagnoses															
Lung		591.97 ± 988.65	118.86 ± 53.98	75.60 ± 91.26	698.02 ± 531.83		660.12 ± 1109.91	115.84 ± 46.30	72.87 ± 59.99	699.19 ± 609.92		0.011	0.015	0.067	0.237
Liver		1275.23 ± 2079.67	124.38 ± 58.28	70.06 ± 53.73	759.04 ± 742.43		1440.39 ± 2518.85	112.14 ± 53.30	72.84 ± 42.55	758.99 ± 678.57		0.030	< 0.001	0.053	0.250
Colorectal		845.64 ± 1374.57	121.60 ± 53.35	73.87 ± 46.87	910.08 ± 896.34		933.59 ± 1648.94	112.52 ± 36.99	72.55 ± 47.04	877.58 ± 797.02		0.057	< 0.001	0.139	0.106
Gastric		977.22 ± 1835.31	140.42 ± 82.31	83.18 ± 110.20	899.97 ± 817.91		714.10 ± 963.80	108.73 ± 24.27	71.69 ± 51.31	762.67 ± 586.45		< 0.001	< 0.001	0.005	< 0.001
Gallbladder, Biliary duct		1043.17 ± 1589.64	122.61 ± 59.19	64.17 ± 52.22	809.09 ± 791.70		874.16 ± 1433.95	109.46 ± 44.84	71.33 ± 63.40	620.69 ± 774.32		0.015	< 0.001	0.009	< 0.001
Pancreas		733.56 ± 1304.37	126.84 ± 58.30	80.97 ± 125.99	689.84 ± 707.61		704.44 ± 977.54	149.48 ± 116.34	70.03 ± 50.63	714.77 ± 596.42		0.179	< 0.001	0.025	0.146
Bladder		942.85 ± 1865.73	120.84 ± 47.70	63.11 ± 42.43	963.38 ± 949.76		804.86 ± 891.96	87.54 ± 7.82	67.50 ± 45.38	777.60 ± 776.42		0.085	< 0.001	0.078	0.008
Prostate		909.24 ± 1329.92	120.44 ± 48.63	64.20 ± 53.11	722.04 ± 659.43		-	-	-	-					
Renal		768.69 ± 1437.47	112.35 ± 43.48	67.65 ± 33.26	738.80 ± 581.23		1149.87 ± 1883.59	118.49 ± 42.35	70.25 ± 43.05	807.47 ± 558.98		0.023	0.072	0.154	0.092
Breast		-	-	-	-		897.04 ± 1508.80	96.73 ± 15.60	65.90 ± 42.75	940.43 ± 1165.51					
Thyroid		587.92 ± 595.33	79.96 ± 1.62	74.88 ± 19.19	1047.16 ± 708.46		764.48 ± 767.95	88.79 ± 8.12	74.31 ± 54.63	468.92 ± 200.62		0.043	< 0.001	0.235	< 0.001
Cervical		-	-	-	-		975.54 ± 1124.55	192.94 ± 204.68	53.68 ± 29.70	724.93 ± 513.60					

*Note.* CPR = cardio-pulmonary resuscitation, MV = mechanical ventilation, ECMO = extra-corporeal membrane oxygenation, HD = hemodialysis

### EOL costs of medical treatments

**Fig. 2:**
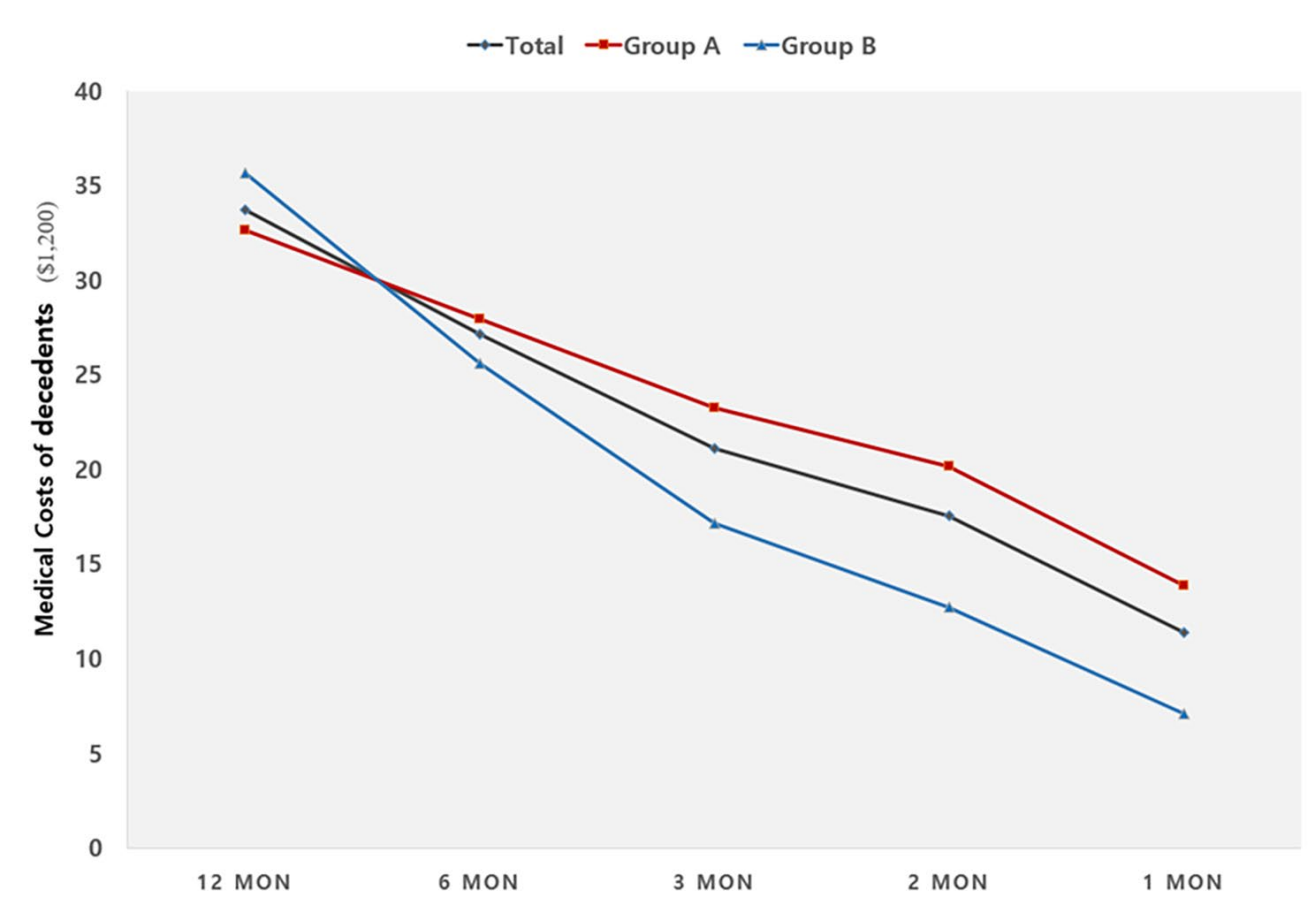
End-of-life costs of medical treatment according to point of time Group A included patients who died during high-intensity treatment in the intensive care unit. Group B included patients who received high-intensity treatment in the intensive care unit, but they did not receive it at the time of death

The total EOL costs of medical treatments in the last year of life among deceased older adults with cancer are presented in Fig. 2. The mean total EOL medical expenses per person for the first year before death was $33,712. The cost of EOL medical expenses for three months and one month before the subjects’ death was $21,117 and $11,389, respectively. They accounted for approximately 62.6% and 33.8% of the total EOL costs during the last year, respectively. Additionally, the costs of EOL medical treatment for groups A and B are shown in Fig. 1. Subjects in Group A (n = 3,221) died while receiving high-intensity treatment in the ICU. Group B (n = 1,403) were those who received high-intensity treatment in the ICU, but did not receive it at the time of death. The cost of total EOL medical treatment spent during the year prior to the subject’s death was higher in group B than in group A (Table 3, *p* < .001). However, costs for 6 months, 3 months, 2 months, and 1 month before the subject’s death were higher in Group A (*p* < .001). The differences in total EOL medical treatment costs between groups A and B increased sharply from 6 months before death, and this difference was maintained until the time of death. In groups A and B, the costs associated with medical treatments during the last month before death were 42.4% and 19.9% of the total EOL expenses during the year, respectively.

**Table 3:** End-of-life costs of medical treatment among deceased older adults with cancer (N = 4,624)

Time point	Total (n = 4,624)	Group A (n = 3,221)	Group B (n = 1,403)	t	p
	M ± SD	M ± SD	M ± SD		
12 mon	33712.09 ± 26162.64	32634.87 ± 26452.39	35676.59 ± 25516.89	-3.360	< 0.001
6 mon	27155.90 ± 21737.33	27987.21 ± 22830.63	25639.85 ± 19502.67	4.945	< 0.001
3 mon	21117.55 ± 17339.84	23288.31 ± 18840.02	17164.10 ± 13332.29	16.736	< 0.001
2 mon	17530.31 ± 14327.33	20162.76 ± 15519.22	12729.78 ± 10237.36	25.899	< 0.001
1 mon	11389.14 ± 10105.83	13841.95 ± 10863.10	7102.51 ± 6747.45	34.607	< 0.001

*Note.* Group A included those who died during high-intensity treatment in the intensive care unit. Group B included those who received high-intensity treatment in the intensive care unit, but they did not receive it at the time of death

## Discussion

This descriptive cohort study utilized national health insurance claims data from South Korea. We investigated medical care costs among older adults with one of the 12 cancers associated with a high mortality rate. Moreover, we examined EOL costs in the last year of life among patients with cancer. Our findings show that the EOL costs of medical treatments spent by older adults increase as the subjects are near the end of their lives.

The elderly population is rapidly growing worldwide as life expectancy increases due to advances in medicine and technology. According to the World Health Organization (WHO), by 2050, it is estimated that approximately one-fifth of the world’s population will be over 60 years old [15]. The pace of population aging in South Korea has also increased dramatically. It is expected that the population aged 65 years or older will account for more than 40% of the total population by 2060 [2]. The life expectancy at birth in 2018 was 82.7 years, while the health-adjusted life expectancy (HALE) was 64.4 years in South Korea [16]. Life expectancy at birth refers to how long a newborn can expect to live, on average, if current death rates do not change [17]. HALE represents the number of years in full health that an individual can expect to live given the current morbidity and mortality conditions [18]. Many people want to live a long and healthy life. However, statistics indicate that we may live with disease for about 20 years from the age of 65 to the end of our life.

As the aging population progresses, the elderly population with cancer is increasing, and the financial burden caused by cancer is also expected to increase. According to statistics in South Korea [19], the population aged ≥ 60 years (56.5%) received more cancer-related treatments compared to subjects of other age groups (2.5–21.6%) in 2019. In cohort studies analyzing data from cancer patients [3, 20], the mean monthly inpatient costs for acute myeloid leukemia (AML) increased from $5,465 12 months before death to $15,033 in the last month [3]. Additionally, approximately 70% of the total medical expenditure during the last year of life among cancer patients was spent in the last 6 months [20]. The pattern of increased EOL care costs was similarly observed in other countries studies [21–23]. Substantial groups of severely ill deceased patients underwent intensive medical treatment until shortly before death in Switzerland [21]. Non-Hispanic (NH) Asian, NH black, and Hispanic patients with lung cancer were likely to receive intensive care in their final month of life in the United States [23]. Furthermore, the Australian cancer cohort had significantly higher rates of health service use and 27% higher total healthcare costs than those without a cancer history [22]. These findings suggest that the medical expenses during EOL of elderly cancer patients might be substantial, which can place a great burden on the national medical finances.

Our findings indicated the total medical treatment costs and high-intensity care costs incurred during the recent ICU admissions among the older population with cancer. The costs for high-intensity care accounted for 6.4% ($849.30) of the total medical treatment costs. High-intensity care costs seem to account for a small proportion of total treatment costs. However, the actual cost associated with high-intensity care is expected to be much higher, considering that cancer is a chronic disease and can lead to repeated ICU admissions in severe cases. Additionally, this study might partially analyze medical costs because the HIRA dataset included only cost information with codes for specific treatment or procedures. According to our previous analysis work [14], the annual cost of high-intensity care has increased steadily from 2016 to 2019. Considering that 95% of inpatient treatment costs are covered by national health insurance when a subject is diagnosed with registered cancer in South Korea [24], high-intensity medical expenditures related to cancer can be a financial burden on the health care system.

In this study, the total medical care costs and costs of high-intensity treatment varied according to age and sex. The costs of medical treatments for those aged 65–69 years were significantly higher than those aged > 85 years. A similar trend has been observed in other studies [22, 25]. Deceased elderly aged over 90 years at death had 20% lower health care costs than those aged 80–84 years in Australia [22]. In a Taiwanese national cohort study of elderly patients (≥ 60 years) with chronic kidney disease [25], the care costs were lower among subjects with advanced ages: every 10-year increase in age was associated with a 3% reduction in 30-day EOL inpatient expenses. Additionally, our results showed differences in medical treatment costs according to sex. The total medical treatment cost was higher in men, whereas the cost of high-intensity treatment was higher in women. These results are consistent with those of Chen and colleagues [25], who reported in Taiwanese study that EOL medical expenses were higher among women (3%; 95% CI, 0–5%). The cost of transfusion was higher in women under 80 years than in men of the same age in our study. This might be one of the factors related to the difference in high-intensity care costs between men and women. However, it is difficult to compare these results, as there are few studies that present the costs of various types of high-intensity treatment and analyze the difference in medical costs between men and women. In addition, the number of men in all cancers was high because men’s prevalence rates are higher than women in all cancer.

Additionally, this study provides recent evidence on the high costs of EOL cancer care among older adults that continue to increase until the last month of life. The total medical expenses were approximately $32,600 for the last year of life, and the cost incurred during one month before death was about one-third of the total EOL cost. According to study by Park and Song [3], the monthly inpatient costs of cancer patients increased steeply from 2 months prior to death between 2013 and 2014 in South Korea. These findings indicate that many Korean elderly cancer patients are likely to receive invasive and aggressive treatment, even to the point of imminent death. The trend of high-intensity care costs is similarly observed in cohort studies in other countries such as the US [26, 27], Canada [8], and Taiwan [28]. EOL care costs during the final 4 months of life were about $10,000 higher for cancer patients than for those without cancer in the US [26], and cancer patients were more likely to use intensive inpatient treatments ($23,938 vs. $17,856). In a Canadian cohort study [8], acute care costs increased rapidly in the last 120 days. Likewise, the EOL care expenditure in cancer decedents was highly concentrated in the last few months in Taiwan [28]; total EOL care expenditures incurred in the last 1, 3, and 6 months were 32.9%, 52.2%, and 72.5%, respectively. Based on these findings, it can be seen that EOL care expenditures associated with high-intensity treatment for older populations with cancer are highly concentrated in the last month.

Medical expenses spent in the last year of the subject’s life indicate the intensity of EOL treatment. This study suggests the likelihood of unnecessary and futile medical spending at the EOL. In a longitudinal multi-institutional cohort previous study [29], patients who had discussed their wishes for EOL care with physicians were more likely to receive care that was consistent with their preferences. The result showed that patients’ preferences not only determine the direction of treatment, but also there is a clear difference in medical costs. Among patients who received no life-sustaining treatments, physical distress was lower in patients for whom such care was consistent with their preferences. Furthermore, a cancer patient’s documenting preferences against resuscitation were associated with better quality of life in the week before death [30]. With the implementation of the LST Decision Act [13] since 2018, cancer patients at the terminal stage have the right to express their intentions regarding EOL medical treatments to their physicians and receive legal guarantees in South Korea. Accordingly, the public has become highly interested in the quality of care at the EOL. To avoid the financial pressure of aggressive EOL care, physical suffering, emotional burden, and failed expectations for care, policy makers and healthcare providers need to focus on the controllable causes that influence accelerating futile EOL care expenditures.

This study is limited in that the HIRA database included only medical care services that were reimbursed by the national health insurance system. Medical services that were paid by patients outside the pocket were excluded from the analysis. Moreover, the HIRA dataset included limited clinical information, such as the patient’s clinical exam results, which can influence cancer care costs. In addition, HIRA database contain only codes and costs of medical service, and not contain the number of cases consulted overall. This study only included information on older adults with cancer treated at tertiary hospitals, so it may not be representative of EOL cancer patients in South Korea. Despite these limitations, this study is significant in that it provides preliminary data to understand the current status of medical care costs at the EOL for cancer patients. Future research should focus on investigating the clinical factors that influence cancer care costs and examining total medical expenditure, including non-reimbursable services costs, to estimate the entire economic burden of cancer care. In the end, the cost of EOL, not the treatment of cancer, is the burden of the state. Therefore, it is necessary to carefully analyze the benefits and disadvantages perform active treatment when the patient is unlikely to recover. Eventually, rather than actively treating terminal cancer patients at the end, it is helpful to consult with the patient in a close interview with the patient and the family. The hospice system will help to settle. However, the purpose was to provide basic data through the analysis of the current EOL status of this study, it is necessary to further study.

## Conclusion

Findings indicate that EOL care for older populations with cancer is highly concentrated until impending death. Medical care costs incurred in the last month accounted for one-third of the total EOL expenditure. Futile high-intensity treatments at the EOL not only place physical, psychological, and economic burdens on patients and their families, but also increases the financial burden on the national health care system. The intensity of EOL care is an important and challenging issue in terms of care quality and cost suitability. Continuous assessment of the appropriateness of medical resource use at the EOL is required to reduce undue burden on the patient, families, and insurance system and to provide optimal care.

## Data Availability

The datasets used and/or analysed during the current study are available from the corresponding author on reasonable request.

## Abbreviations

AML: Acute myeloid leukemia
CPR: Cardiopulmonary resuscitation
ECMO: Extracorporeal membrane oxygenation
EOL: End-of-life
GDP: Gross domestic product
HALE: Health-adjusted life expectancy
HD: Hemodialysis
HIRA: Health Insurance Review and Assessment Services
ICD-10: International Classification of Disease-10th revision
ICU: Intensive care unit
KNHI: Korean National Health Insurance
MAP: Medical Aid Program
MV: Mechanical ventilation
NH: Non-Hispanic
OECD: Organization for economic co-operation and development
SD: Standard deviations
WHO: World Health Organization
